# Quality, Functionality, and Features of Chinese Mobile Apps for Diabetes Self-Management: Systematic Search and Evaluation of Mobile Apps

**DOI:** 10.2196/14836

**Published:** 2020-04-07

**Authors:** Enying Gong, Zongmuyu Zhang, Xurui Jin, Yishan Liu, Lumin Zhong, Yao Wu, Xuefeng Zhong, Lijing L. Yan, Brian Oldenburg

**Affiliations:** 1 Melbourne School of Population and Global Health University of Melbourne Melbourne, Victoria Australia; 2 Global Health Research Center Duke Kunshan University Kunshan, Jiangsu China; 3 School of Medicine Tsinghua University Beijing China; 4 School of Public Health Shanghai Jiaotong University Shanghai China; 5 College of Science and Engineering University of Edinburgh Edinburgh United Kingdom; 6 Department of Epidemiology and Public Health University College London London United Kingdom; 7 Melbourne School of Engineering University of Melbourne Melbourne Australia; 8 Department of Personal Health Philips Research China Shanghai China

**Keywords:** diabetes mellitus, self-management, mobile apps, China

## Abstract

**Background:**

The emergence and advancement of mobile technologies offer a promising opportunity for people with diabetes to improve their self-management. Despite the proliferation of mobile apps, few studies have evaluated the apps that are available to the millions of people with diabetes in China.

**Objective:**

This study aimed to conduct a systematic search of Chinese mobile apps for diabetes self-management and to evaluate their quality, functionality, and features by using validated rating scales.

**Methods:**

A systematic search was conducted to identify Chinese apps for diabetes self-management in the four most popular Chinese language mobile app stores. Apps were included if they were designed for diabetes self-management and contained at least one of the following components: blood glucose management, dietary and physical activity management, medication taking, and prevention of diabetes-related comorbidities. Apps were excluded if they were unrelated to health, not in Chinese, or the targeted users are health care professionals. Apps meeting the identified inclusion criteria were downloaded and evaluated by a team of 5 raters. The quality, functionalities, and features of these apps were assessed by using the Mobile App Rating Scale (MARS), the IMS Institute for Healthcare Informatics Functionality score, and a checklist of self-management activities developed based on the Chinese diabetes self-management guideline, respectively.

**Results:**

Among 2072 apps searched, 199 were eligible based on the inclusion criteria, and 67 apps were successfully downloaded for rating. These 67 apps had an average MARS score of 3.42 out of 5, and 76% (51/67) of the apps achieved an acceptable quality (MARS score >3.0). The scores for the four subdomains of MARS were 3.97 for functionality, 3.45 for aesthetics, 3.21 for information, and 3.07 for engagement. On average, reviewed apps applied five out of the 19 examined behavior change techniques, whereas the average score on the subjective quality for the potential impact on behavior change is 3 out of 5. In addition, the average score on IMS functionality was 6 out of 11. Functionalities in collecting, recording, and displaying data were mostly presented in the reviewed apps. Most of the apps were multifeatured with monitoring blood glucose and tracking lifestyle behaviors as common features, but some key self-management activities recommended by clinical guidelines, such as stress and emotional management, were rarely presented in these apps.

**Conclusions:**

The general quality of the reviewed apps for diabetes self-management is suboptimal, although the potential for improvement is significant. More attention needs to be paid to the engagement and information quality of these apps through co-design with researchers, public health practitioners, and consumers. There is also a need to promote the awareness of the public on the benefit and potential risks of utilizing health apps for self-management.

## Introduction

### Background

Diabetes is a complex chronic disease that affects more than 100 million individuals in China [1]. The prevalence of diabetes was estimated to be 10.9% in China in 2013 [2], whereas the overall awareness, treatment, and control rates were 38.6%, 35.6%, and 33.0%, respectively [3]. Many challenges exist regarding the provision of high-quality care for patients with diabetes and improving the control rate at the population level. Effective management and control of diabetes require intensive long-term efforts from patients and health care providers. Although primary health care services related to diabetes care are 1 of the 14 items of the Basic Public Health Service in China [4], access to high-quality health services is still quite limited. In addition, low health literacy and lack of awareness on appropriate self-management strategies have further restrained the effective control among people with diabetes [5]. Therefore, developing low-cost and effective strategies for improving the self-management of diabetes is essential.

The emergence and advancement of mobile health (mHealth), defined as the use of mobile technologies for improving health care processes and outcomes, offer a promising opportunity for people with diabetes to improve their self-management and health outcomes [6-8]. Mobile phone usage among Chinese adults is now almost ubiquitous (96.8 mobile cellular subscriptions per 100 people) [9], and the number of people who surf online through their phones reached 800 million in China in 2018 [10]. Smartphone penetration accounts for 48% of all phone users [11], and there are more than 4 million mobile apps already available in the market [10]. This widespread dissemination of mobile technologies creates a huge opportunity to transform health care delivery in China. Although the evidence remains inconclusive, empirical studies have shown that mHealth interventions have the potential to improve patients’ access to low-cost care, facilitate patient-provider communication, and make an impact on patients’ health outcomes and quality of life [12-15].

The number of publicly available mobile apps related to diabetes and diabetes self-management has grown exponentially over the last 10 years in the global markets [8,15]. Previous studies examined English apps targeted on diabetes self-management from various dimensions, including the quality, the functions, the usability, and the application of behavior change techniques, and found that the quality and performance of these apps in general were suboptimal [8,15-19]. A few studies reviewed mobile apps for chronic disease management in China in general [20-22], but these reviews were not able to provide a comprehensive assessment of the quality of the existing mHealth solutions for people with diabetes in China.

### Objectives

To address this gap, the main aim of this study was to conduct a systematic review and evaluation of mobile apps for diabetes self-management in China. The specific objectives were (1) to provide an overview of the available Chinese mobile apps for diabetes self-management, (2) to evaluate the quality of these apps with validated rating scales, and (3) to describe the key functions and features of these apps in helping people with diabetes. The study was conducted by following the Preferred Reporting Items for Systematic Reviews and Meta-Analyses framework [23].

## Methods

### Systematic Searching and Screening

We conducted a systematic search of mobile apps related to diabetes self-management from four Chinese language mobile app stores from January 2018 to March 2018. On the basis of the popularity of the app stores, we identified four app stores as target platforms, including Apple iTunes Store for the iPhone operating system (iOS) as well as Tencent Myapp, 360 Mobile Assistant, and Baidu Mobile Assistant for the Android system. According to a preliminary estimate, these three Android stores accounted for more than half of the market share of the Android app stores in China [24]. Keywords for searching include diabetes, blood glucose, diabetes prevention, diabetes control, and diabetes treatment. Each keyword was searched through the general search bar of the four app stores listed above.

The eligibility screening was conducted by 2 reviewers (EG and ZZ). The duplicate apps yielded from multiple stores or multiple searching terms were removed from the pool. Then, the 2 reviewers checked the eligibility of the apps based on the titles, descriptions, and screenshots shown in the app stores. Apps were included if they were used for diabetes self-management and contained at least one of the following components: blood glucose management, dietary and physical activity management, medication taking, and prevention of diabetes-related comorbidities. Apps were excluded if they were unrelated to health, not in Chinese, or the target users are health care professionals. After two rounds of screening, a list of apps was generated for further download and evaluation.

### Evaluation and Data Extraction

A team of 5 raters downloaded the screened apps and independently tested the quality, functionality, and features of the apps from August 2018 to October 2018. All raters attended a workshop for this project and received formal training regarding the study protocol and evaluation instruments, read through the handbook, and passed a pilot test before formally rating the apps. For each app reviewed, raters downloaded the app on their phone and used all the functions of the app to familiarize themselves with the app before conducting the rating. Raters then went through all the questions in the data extraction form and performed the rating. Moreover, more than 10% (8/80) of the apps were randomly selected and double rated to check their inter-rater reliability. Differences were discussed to examine whether these differences existed between systems or in the evaluation.

### Rating Instrument and Measurement

The data extraction form was developed based on the Qualtrics online platform (Qualtrics). The form included four parts: (1) general information of the app, (2) the quality of the app based on the Mobile App Rating Scale (MARS) [25], (3) the scope of functionality of the app based on the IMS Institute for Healthcare Informatics Functionality score [26], and (4) a checklist regarding the features and contents of the app.

#### General Information

The general information section primarily extracted data that could be found from the description of the app in the app stores, including app name, operating platform, developer, version, date of the recent update, and cost. The star rating score and the number of raters were extracted based on the iOS app market because of the largely unavailable star rating in the Android stores and the diverse scale range applied across the three Android stores. The number of downloads was only available in the Android app store, and the statistics from each store were recorded if available. In addition to these descriptive information shown in the app stores, technical aspects of the app (eg, allowing password protection and requiring log-in) and 19 behavior change techniques (eg, assessment, feedback, information or education, monitoring, advice, and goal setting) were also collected based on the checklist of MARS [25] and previous studies [27].

#### Quality

The quality of each app was evaluated by using MARS, which is a simple, reliable, objective rating tool to provide a multidimensional measure of the app quality [25]. The scale has been shown to have excellent internal consistency and inter-rater reliability in previous studies [28]. The scale contains 19 items grouped into four domains, including engagement (entertainment, interest, customization, interactivity, and target group), functionality (performance, ease of use, navigation, and gestural design), aesthetics (layout, graphics, and visual appeal), and information quality (accuracy of app description, goals, quality and quantity of information, visual information, credibility, and evidence base). Each item was measured on a 5-point Likert scale, with 1 indicating inadequate and 5 indicating excellent. A mean score for each domain and a mean score for overall 19 items were computed as the score for the quality of the app. In addition to the objective assessment, five items were used to assess the subjective quality in terms of the perceived impact of the app on users’ knowledge, attitude, and intention to change and the likelihood of actual change in diabetes self-management. These subjective quality items were scored separately.

#### Functionality

The functionality of the app was measured by using the IMS functionality score [26]. Unlike the functional domain within the MARS that reflects whether the app functions well, the IMS functionality score focused on the scope of the functions. The score contains seven functionality categories (informing, instructing, recording, displaying, guiding, reminding, and communicating information) and four subcategories (collecting data, sharing data, evaluating data, and intervening). Apps allowing the function were coded as 1, otherwise coded as 0. A functionality score ranging from 0 to 11 was generated for each app.

#### Features and Contents

For each app, we also evaluated its features and contents in promoting diabetes self-management activities. A checklist, including health indicators and behaviors monitoring and reminding, health education, and communication with professionals and peers, was derived from previous studies [8,17] and the Chinese diabetes self-management guideline [29].

### Quality Control

To ensure the quality of the study, fidelity, and consistency of ratings among the raters, a handbook for the raters was developed, reviewed, and refined by experts before implementing the research activities. A workshop was undertaken in China in March 2018 to review and finalize the study protocol and the handbook.

### Statistical Analysis

All the information collected through the Qualtrics online platform was downloaded for further analysis. Descriptive analysis was conducted, and the mean and SD were reported. If the data distribution was skewed, the median and IQR were reported. The inter-rater reliability score was calculated between two records generated from the double rating [28]. All analyses were conducted using Stata statistical software, version 14 (StataCorp LP), and the visualized figures were drawn using Excel (Microsoft Excel for Office 365).

## Results

### Systematic Search and Screening

**Figure 1:**
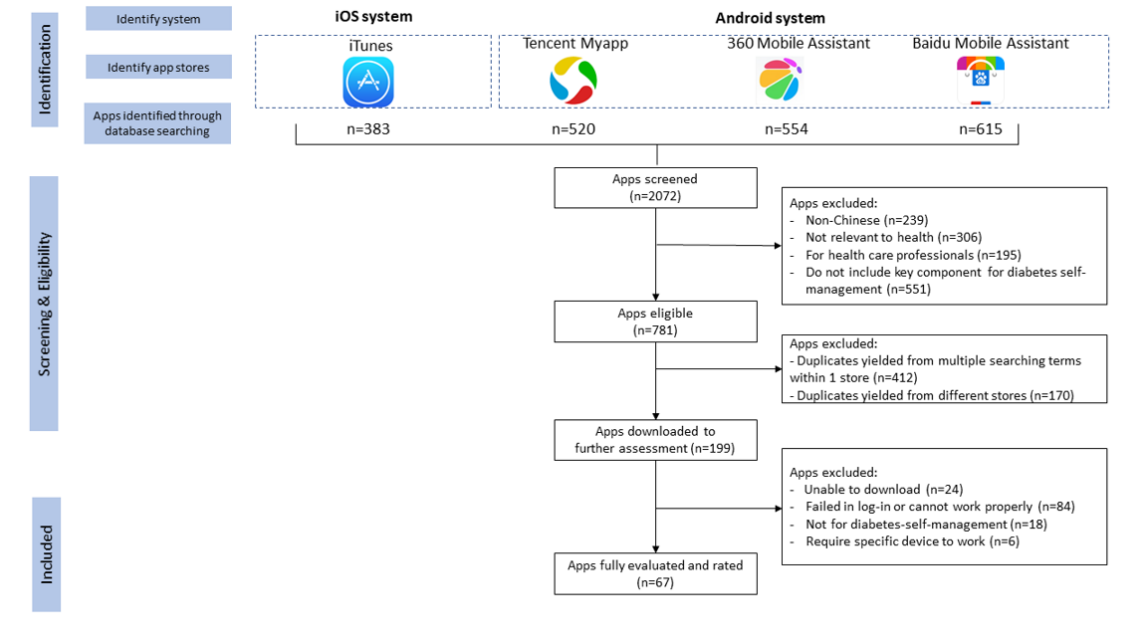
Screening process based on the Preferred Reporting Items for Systematic Reviews and Meta-Analyses diagram.

A total of 2072 apps were identified from the initial search in four Chinese language app stores. After excluding the duplicates, 936 apps were enrolled for eligibility screening, and 199 of these apps met the inclusion criteria for further download and evaluation. However, among these apps, 108 were failed in download or registration or did not work properly after download. Moreover, 18 apps were excluded because the raters found that these apps did not meet the inclusion criteria on diabetes self-management, and another six apps were excluded because they had to be linked with specific devices to operate. Finally, 67 apps met the inclusion criteria and were formally evaluated. Figure 1 provides an overview of the screening process.

### Characteristics of the Apps

Multimedia Appendix 1 provides a list of the included apps and their characteristics. Moreover, 41 apps had both Android and iOS versions, 25 apps could run only on Android phones, and one app was only available in the iOS market. As for the developer of these apps, 24 apps were developed by informatic or internet technology companies, 38 by health management or biomedical companies, one by a pharmaceutical company, and three involved clinical institutions or science institutions as their codevelopers. About half (33/67 49%) of these apps released the latest version in 2018. All apps involved in the review were free to download.

On the basis of the statistics in the Android markets, the median number of downloads was 15,000 (IQR 1025-330,000), 11,000 (IQR 446-78,000), and 20,000 (IQR 1000-180,000) for Baidu, Tencent, and 360 app stores, respectively, and one app reached 7.7 million downloads as the largest number across the three stores, despite these number of downloads not necessarily implying the real users who suffered from diabetes. In addition, 73% (30/41) of apps in the iOS market had the star rating score available, with a median rating score of 4.7 (IQR 4-5), and the median number of raters was 54 (IQR 12-186). Of 67 apps, 48 (72%) required setting an account and logging in before using, and 40 (60%) allowed password protection.

### Presence of Behavior Change Techniques

The average number of behavior change techniques used in the reviewed apps was 5 (SD 3.2) out of the 19 behavior change techniques assessed. The most frequently identified behavior change technique was self-monitoring or tracking contained in 87% (58/67) of apps, followed by information or education in 64% (43/67) of apps, assessment in 60% (40/67) of apps, feedback in 57% (38/67) of apps, and advice, tips or strategies in 54% (36/67) of apps.

Multimedia Appendix 2 presents the number of apps that applied each of the behavior change techniques.

### App Quality

**Figure 2:**
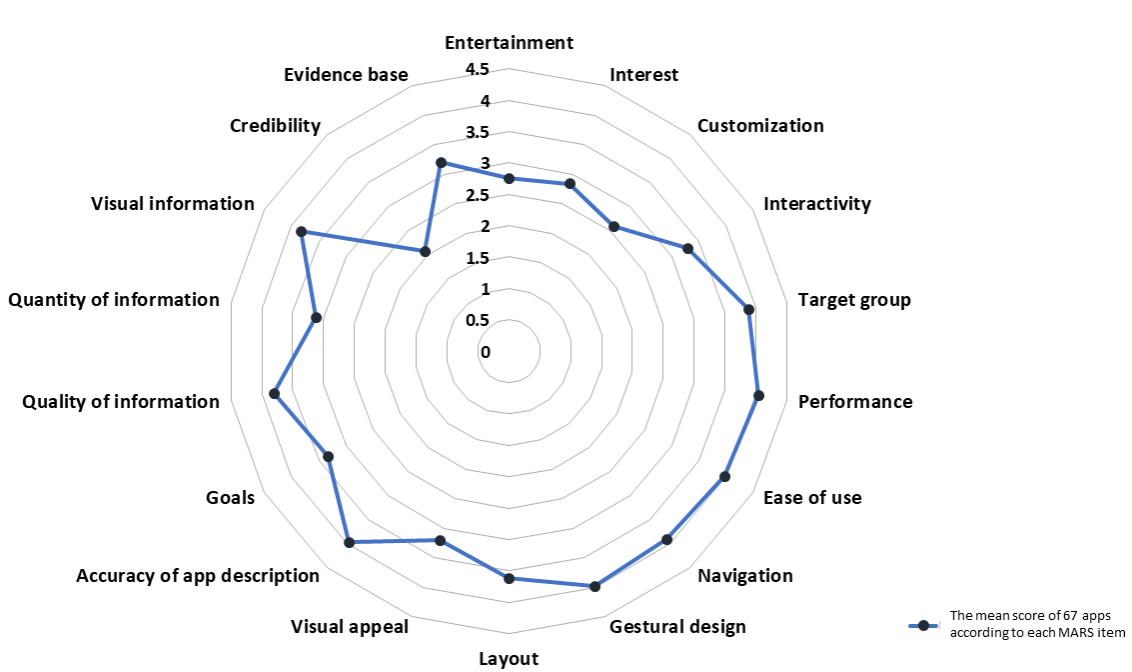
The average score of each Mobile App Rating Scale item.

The average MARS score of all apps reviewed was 3.42 (SD 0.66). A total of 76% (51/67) of apps had a minimum score of 3.0, indicating that these apps reached an acceptable quality level. As for the four domains, functionality had the highest score with an average of 3.97 (SD 0.66), followed by aesthetics (mean 3.45, SD 0.88), information (mean 3.21, SD 0.73), and engagement (mean 3.07, SD 0.90). Figure 2 presents the mean score for each MARS item. Apps received a higher score in the items of performance, gestural design, ease of use, navigation, and accuracy of app description, whereas the score on credibility, customization, entertainment, and interest were relatively low. For item 19, only two apps were tested with quasi-experimental trials to evaluate their efficacy, despite the weakness of the study design in sample size and follow-up period [30,31]. Eight apps were double rated and reached an inter-rater reliability score of 0.56 (95% CI 0.41-0.69).

The results of the subjective measurement of app quality are presented in Table 1. Raters evaluated that only three apps were likely to attract frequent users. Although all apps were free, seven apps were rated as “willing to pay” by raters. In terms of the perceived impact of these apps in promoting behavior change, apps reached an average score of 3.0 (SD 0.9) for five subjective items of MARS. Raters strongly agreed or agreed that half of these apps might improve users’ awareness and knowledge, but only 30% (20/67) of the apps had the potential to change users’ attitudes, intention to change, and lead to the actual change in diabetes self-management and related health outcomes.

**Table 1:** Subjective measurement of quality of apps (N=67).

Subjective quality statement		Apps, n (%)
**Recommend apps to people who might benefit from it**		
	Not at all	23 (34)
	A few people	16 (24)
	Several people	16 (24)
	Many people	10 (15)
	Everyone	2 (3)
**Times of use of this app in the next 12 months if the app is relevant**		
	None	24 (36)
	1-2	17 (25)
	3-10	13 (19)
	10-50	10 (15)
	>50	3 (5)
**Willing to pay for the app**		
	No	40 (60)
	No or probably not	9 (13)
	Maybe	11 (16)
	Probably yes	7 (11)
**Strongly agree or agree that the app will improve**		
	Awareness	35 (52)
	Knowledge	36 (54)
	Attitude	22 (33)
	Intention to change	26 (39)
	Behavior change	26 (39)

### Functionality

**Figure 3:**
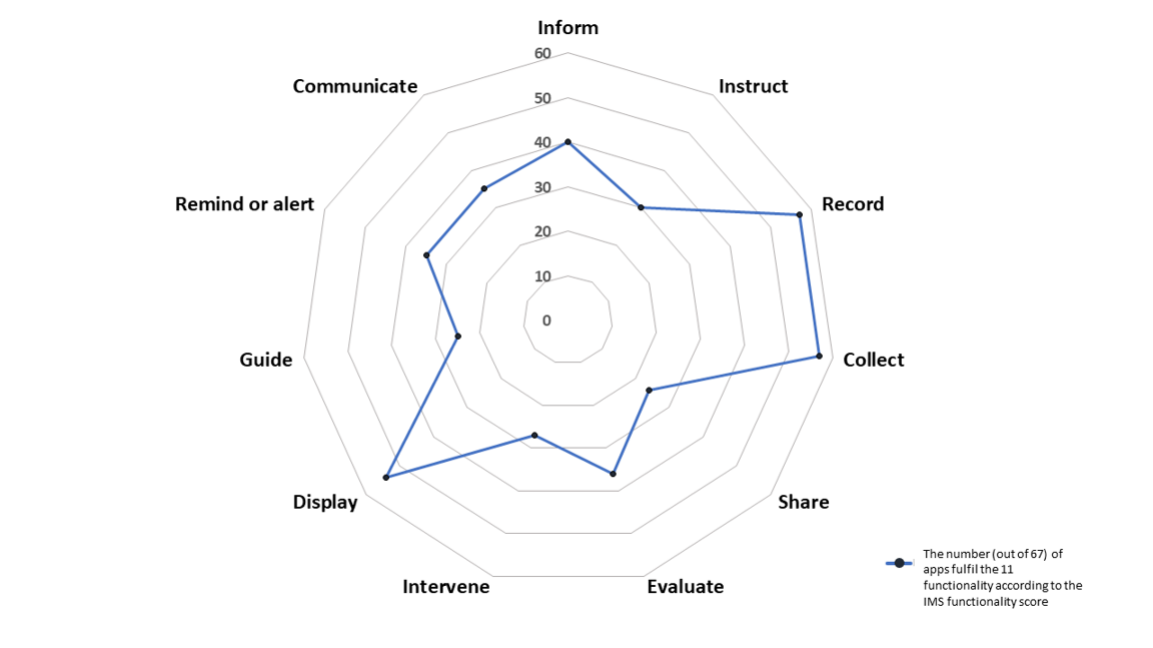
Number of apps that meet the functionality categories.

Figure 3 presents the number of apps that meet the functionality category based on the scale. On average, the apps reviewed met 6.3 functionalities out of the 11 items of the scale (SD 2.8). Moreover, four apps had all 11 functionalities, and five apps had 10 functionalities. Among the 67 apps, 57 (85%) had the function to capture user-entered data and store the data on the user’s phone, and 54 (80%) apps could graphically display user-entered data. However, only about half of the reviewed apps (36/67, 54%) were able to evaluate the entered data, 36% had the function to share and transmit the entered data, and 40% (27/67) were able to promote intervention based on the data collected. More than half of the apps had the function to provide information, instruction, and guidance to the users. Among the 67 apps reviewed, 35 apps provided support for communication with others.

### Features and Contents

All the reviewed apps contained multiple features to fulfill users’ activities in self-management (Table 2). Among all features, tracking and monitoring health conditions and lifestyle behaviors was the most popular one that presented in most of the apps. About 81% (54/67) of the reviewed apps fulfilled the users’ needs in monitoring blood glucose, but less than half of these apps supported data transfer from wearable or medical devices. Setting individual reminders on self-management–related activities is another key feature in reviewed apps, and blood glucose testing (23/67 34%), medication taking (21/67 31%), and blood pressure testing (11/67 16%) were the most common purpose of reminders. In addition, apps also provided health education information in text, pictures, and videos and covered a broad range of topics, such as blood glucose control and diet (in 44/67, 66% apps), diabetes-related concepts (in 39/67, 58% apps), and physical activities (in 36/67, 54% apps). However, stress and emotional control and blood cholesterol control only presented in about one-fourth of the reviewed apps. In addition, less than 10% (7/67) of the apps were able to provide individualized health education information to meet the needs of users with various health literacy levels.

In addition to self-monitoring and health education, these apps also supported users to connect with health professionals and other patients online or offline. Among 67 apps, 26 apps (39%) supported online consultation with health care professionals, and four apps (6%) helped users make appointments with health care providers offline. Moreover, 11% (7/67) apps assisted users in navigating the closest pharmacies or clinics, and 22% (15/67) contained an online market for purchasing medicines, supplements, or devices. Besides, 33% (22/67) of apps contained social forums or blogs to facilitate communication between peers, and 27% (18/67) of apps supported data sharing with family members.

**Table 2:** Features to support diabetes self-management.

Features		Apps with manual data entry, n (%)	Apps with auto-transfer from wearable/medical devices, n (%)
**Track and monitor health conditions**			
	Blood glucose level	54 (81)	29 (43)
	Blood pressure level	34 (51)	10 (15)
	Blood cholesterol level	10 (15)	2 (3)
	Physical activity level	34 (51)	13 (19)
	Diet pattern	26 (39)	0 (0)
	Weight	33 (49)	7 (11)
	Medication taking	24 (36)	0 (0)
**Health education on**			
	Information about diabetes	39 (58)^a^	7 (11)^b^
	Blood glucose control	44 (66)^a^	8 (12)^b^
	Blood pressure control	22 (33)^a^	2 (3)^b^
	Blood cholesterol control	15 (22)^a^	0 (0)^b^
	Physical activity	36 (54)^a^	5 (8)^b^
	Healthy diet	44 (66)^a^	5 (8)^b^
	Weight control	26 (39)^a^	1 (2)^b^
	Diabetes-related complications	35 (52)^a^	6 (9)^b^
	Medication use and adherence	30 (45)^a^	7 (11)^b^
	Stress and emotional control	17 (25)^a^	1 (2)^b^
**Set individualized reminders on**			
	Blood glucose testing	23 (34)	N/A^c^
	Blood pressure testing	11 (16)	N/A
	Physical activity	10 (15)	N/A
	Healthy diet	7 (11)	N/A
	Weight test	7 (11)	N/A
	Medication taking	21 (31)	N/A
	Appointment with physicians	5 (8)	N/A
**Make an appointment with physicians**			
	Online consultation	26 (39)	N/A
	Face-to-face consultation	4 (6)	N/A
**Share recorded data**			
	With health professionals	24 (36)	N/A
	With family members/friends	18 (27)	N/A
Communicate through forums/blogs		22 (33)	N/A
Evaluate the risk of having complications		17 (25)	N/A
Purchase medicines/devices		15 (22)	N/A
Find out pharmacy stores/clinics		7 (11)	N/A

^a^General information.

^b^Individualized information.

^c^Not applicable.

## Discussion

### Principal Findings

In this study, we provide a snapshot of publicly available mobile apps for diabetes self-management in China. Through the searching and screening of more than 2000 apps, 67 apps were identified as being suitable for an in-depth evaluation. On the basis of a comprehensive review, our study found that the quality of these apps is suboptimal with considerable variability. Although most of the reviewed apps have multiple features, some common deficiencies were identified as poor engagement, low adherence to guidelines, and lacking evidence on health benefits.

We observed a consistent pattern with previous reviews showing that most of the apps performed better in the domains of functionality and aesthetics but poorly in information and engagement [18,27,32]. Although studies have shown that engagement is crucial for the users’ uptake and the improvement in patients’ health outcomes [33], most of the apps that we reviewed were not able to apply effective strategies to improve users’ experience and engagement. For example, wireless sensors are now widely available, but less than half of the apps that we reviewed supported wireless automatic data acquisition for blood glucose monitoring. Manual data input exposes the users to erroneous workload, which may lead to poor engagement, low compliance, and abandoning [17].

In addition to the issue of engagement, the quality and evidence base of information provided by the apps could also be improved. Compared with previous studies that suggested insufficient health education features within apps [17], our review showed a higher proportion of apps designed with health education features. However, the source of information in some of the reviewed apps was not able to be identified and likely not to be evidence-based. The absence of such evidence-based information may expose end users to misleading or incorrect information, thus putting people with diabetes at risk of potential negative health outcomes. Another critical issue is that only a small proportion of the reviewed apps could set personalized reminders or provide personalized information to users. Although the advantages of using the mHealth tools in providing a large amount of tailored information in real time have been well acknowledged [34], most of the apps involved in our review were not able to achieve the expectation of providing patient-tailored support and health education.

It is also important to note that most of the reviewed apps only partially meet the requirements recommended by the clinical guidelines for diabetes self-management [29]. Supports in managing diabetes-related comorbidities and complications were unavailable in about half of the reviewed apps. For example, activities such as tracking blood pressure and blood cholesterol, stress management, emotional control, frequent foot check, and eye tests were absent in most of the apps. This result is consistent with previous studies that focused on the adherence to guidelines of diabetes apps [16,20]. As most of these apps were developed by informatics or health care technology companies, the involvement of health professionals with a deeper understanding of the best clinical guidelines was insufficient. Therefore, the public, researchers, and policymakers should be aware of the limitations and potential risks of the currently available apps. A multidisciplinary development and co-design process with extensive involvement from health professionals should be highly recommended for future app design.

Another emergent finding from this study was that about 40% (27/67) of the reviewed apps provided an online consultation to users and supported offline appointment making, and 5% (34/67) of the apps contained features to link the online consultation to the offline health care services. This feature had not been identified in previous reviews of English apps. As China faces significant challenges in addressing the increasing burden of chronic diseases, online consultation and telemedicine have been considered as an innovative approach with the potential to provide high-quality, accessible, and affordable care beyond geographical boundaries. Since April 2018, the Chinese government has promoted the “Internet plus healthcare” initiative as a strategy to alleviate the problem of inaccessible and expensive health care services and encourage online health care services, including consultation, appointment making, and test result inquiry [35]. Therefore, we observed an increasing number of apps containing features of online consultation or linking online services with offline care. However, only a few reviewed apps support the data sharing between patients and providers, let alone linking the data in the apps with existing health care records. This weakness in current apps indicates that further efforts are needed to integrate such services with the existing health care information system.

The actual impact of these apps in supporting users’ behavior change and improving diabetes-related health outcomes is unclear. Although a variety of behavior change techniques were incorporated into many of these apps, most of these techniques were applied to support information exchange and records, such as monitoring, education, and assessment. Consistent with the findings from previous studies [36-38], behavior change techniques, as internal drivers, were applied only in a few apps, and the techniques that could provide just-in-time intervention for behavior changes were underutilized. In addition, only two apps in the review had been tested through quasi-experiment trials to evaluate its efficacy on health outcomes [30,31]. Similar to most of the studies that examined the effectiveness of apps for diabetes self-management [12,15], these two studies had a relatively small sample size and were unable to observe meaningful long-term effects. Therefore, the effectiveness and benefit of using these apps for diabetes self-management need to be further examined.

### Comparison With Other Research

To the best of our knowledge, this is one of the few studies that utilized validated scales for evaluating Chinese language mobile apps. Although there are previous studies that reviewed Chinese language apps for either chronic disease in general [22] or diabetes [20,21], our study has a broader scope and utilized validated scales. The four most popular Chinese language mobile app markets were selected as the searching source, which ensured the representativeness and high coverage of apps in this review. This study also analyzed the quality, functionality, and features of apps by using validated rating scales, which filled the research gap and makes the comparison across apps possible in the future.

Comparing these results with those obtained from previous reviews of comparable English apps, a similar level of the quality of apps was found [18,27,32,39]. The apps involved in this review also had similar deficiencies in terms of insufficient engagement strategies, lacking patient-tailored evidence-based information, inadequate functions for a variety of self-management activities recommended by the guideline, and the absence of integration with the existing health care system [8,17]. In addition, although there is an increasing number of trials investigating the effectiveness of English mobile apps for diabetes self-management [40,41], only a few such trials have been undertaken in China where there is the largest population of people with diabetes. Future studies are in great need to evaluate the effectiveness of these publicly available apps in improving the health outcomes among people with diabetes.

### Limitations

This study has some limitations. First, the apps were evaluated by researchers based on short-term use. These results, therefore, cannot reflect the opinion of actual target users—people with diabetes in China—who are generally elderly with relatively poor health and digital literacy. Therefore, further research is needed to evaluate these apps by people with diabetes. Second, although we reached good coverage in selecting apps from the four most popular app stores, apps that were not publicly available were not included. For example, apps that were only available to download by private invitation and apps that can only be used with the support of specific medical devices were excluded. Third, some differences in results were observed through the double rating process. On the basis of the standard protocol, the differences between raters were discussed in detail, and it was found that the inconsistency in score could be explained by the differences in the operating systems or the phone model as well as the different information dispatched by the system based on the log-in information. In addition, although the MARS was rigorously developed, tested, and widely applied in evaluating health apps for various conditions worldwide [27,32], there has been no study evaluating the reliability and validity of the scale in evaluating Chinese apps. To minimize the potential issues in adopting the English version of the scale for the Chinese app review, we provided handbooks with detailed explanations and examples for all raters, organized training sessions with pilot testing, and discussed potential issues thoroughly before the rating. Finally, we were not able to evaluate apps in all conceivable dimensions. For example, privacy and information security were other key domains to evaluate health apps, but our study only assessed it through whether the app needed a log-in and allowed password protection. We were also not able to analyze professionally whether the advice provided for users were accurate and evidence-based. Future studies should focus on these aspects of the evaluation.

### Conclusions

With the proliferation of available technologies, mobile apps are promising tools to support diabetes self-management. In general, the publicly available Chinese apps for diabetes self-management involved in the review contained multiple functions and features, but the quality is suboptimal with enormous potential for improvement. More work is needed to improve these apps by applying strategies to engage users, providing more comprehensive and evidence-based information, and to support a broader range of activities recommended by the clinical guidelines. Rigorous scientific evaluations of the effectiveness and value of these apps on behavior change and health outcomes are also greatly needed.

This study also provides important public health implications. The proliferation of smartphones enables the public to access mobile apps as a potential tool for health promotion and disease self-management. However, the public may not be able to fully realize the potential risks of using mobile apps if the apps have not been designed with full adherence to the clinical guidelines or have not been evaluated on its effectiveness. To overcome this challenge and increase the impact of mobile apps on health improvement at the population level, health researchers and professionals should be more engaged in the development and evaluation of the mHealth technologies to increase the quality of available apps and deliver evidence on the effectiveness. In addition, the government should pay attention to the quality and safety of publicly available apps, set up platforms to help health care providers and patients identify evidence-based health apps, and promote health literacy and awareness among the public on the potential benefit and risks of using health apps.

## Appendix

Multimedia Appendix 1 Characteristics of evaluated apps.

Multimedia Appendix 2 Number of apps that applied behavior change techniques.

## Abbreviations

MARS: Mobile App Rating Scale
mHealth: mobile health
iOS: iPhone operating system
