# Supplementary material for: Quality, Functionality, and Features of Chinese Mobile Apps for Diabetes Self-Management: Systematic Search and Evaluation of Mobile Apps
Source: JMIR Mhealth Uhealth. 2020 Apr 7;8(4):e14836. doi: 10.2196/14836 (PMC7175187; doi:10.2196/14836)
Supplement: Multimedia Appendix 1 [file mhealth_v8i4e14836_app1.docx]

**Supplementary Table 1. Characteristics of Evaluated Apps**

| **ID** | **App Logo** | **System platform** | **Date of latest update** | **Version** | **Type of developers** | **Star Rating^¶^** | **number of raters^¶^** | **Number of downloads in App Stores ^§^** | | | **Log-in**  **required** | **Password**  **protected** |
| --- | --- | --- | --- | --- | --- | --- | --- | --- | --- | --- | --- | --- |
|  |  |  |  |  |  |  |  | **Baidu** | **Tencent** | **360** |  |  |
| 1 | 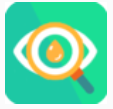 | iOS  & Android | 2017/10/19 | 1.0.1.2 | IT and clinical institution | NA | NA | 800 | 446 | 75 | YES | YES |
| 2 | 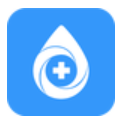 | iOS  & Android | 2018/09/05 | 4.9.6 | Health technology | 4.8 | 81 | 3,000k | 480k | 2,080k | YES | YES |
| 3 | 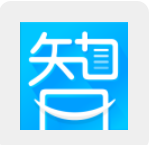 | iOS  & Android | 2018/09/27 | 1.23.3 | Health technology | 3.7 | 12 | NA | 1513 | 66 | YES | YES |
| 4 | 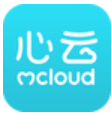 | iOS  & Android | 2018/10/02 | 4.2.15 | Health technology | 4. | 15 | 7,970 | 35k | 20k | YES | YES |
| 5 | 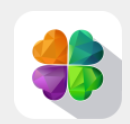 | iOS  & Android | 2018/03/01 | 15.14.55 | Health technology | 5 | 6 | NA | 2577 | NA | YES | YES |
| 6 | 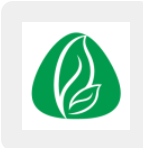 | iOS  & Android | 2018/08/25 | 3.3.4 | Information technology | 3.6 | 27 | 200k | 74k | 11k | YES | YES |
| 7 | 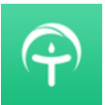 | iOS  & Android | 2018/01/12 | 3.4.2 | Information technology | 4.2 | 5 | NA | 12k | 220k | YES | YES |
| 8 | 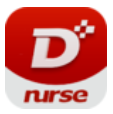 | iOS  & Android | 2018/08/01 | 3.7.6 | Health technology | 4.9 | 1,830 | 800k | 270k | 136k | YES | NO |
| 9 | 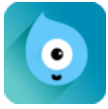 | iOS  & Android | 2018/08/01 | 4.0.0 | Health technology | 4.7 | 124 | 20k | 39k | 10k | YES | NO |
| 10 | 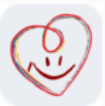 | iOS  & Android | 2018/06/01 | 3.2.4 | Pharmaceutical company | 4.9 | 308 | 1463k | NA | 230k | YES | NO |
| 11 | 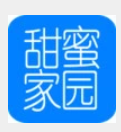 | iOS  & Android | 2017/05/18 | 1.0.57 | Information technology | 3.7 | 7 | 100k | 340k | 40k | YES | YES |
| 12 | 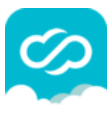 | Android | 2018/10/25 | 2.4.6 | Health technology | NA | NA | 240k | 20k | 130k | YES | YES |
| 13 | 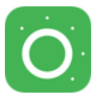 | iOS  & Android | 2018/02/01 | 5.1.0 | Information technology | 4.4 | 27 | NA | 620k | 1,130k | YES | YES |
| 14 | 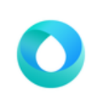 | iOS  & Android | 2017/07/09 | 5.9.1 | Health technology | NA | NA | 40k | 50k | 40k | YES | YES |
| 15 | 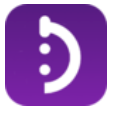 | iOS  & Android | 2018/02/13 | 4.5.3 | Information technology | 5 | 3,610 | 7,790k | 1,255k | 1,747k | YES | YES |
| 16 | 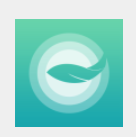 | Android | 2016/09/18 | 1.0 | Health technology | NA | NA | 275 | 35 | NA | YES | YES |
| 17 | 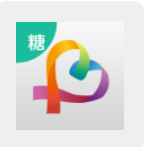 | iOS  & Android | 2015/05/18 | 3.6.0 | Information technology | 4.5 | 134 | 365 | 123 | NA | YES | YES |
| 18 | 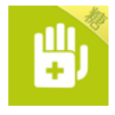 | iOS  & Android | 2018/05/01 | 5.0.22 | IT and clinical institution | 4.5 | 134 | 1,230k | 440k | 1,120k | NO | YES |
| 19 | 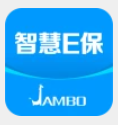 | iOS  & Android | 2018/09/03 | 4.1.20 | Health technology | 3.1 | 125 | 330k | 490k | 90k | NO | YES |
| 20 | 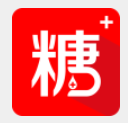 | iOS  & Android | 2018/03/01 | 3.1.2 | Information technology | NA | NA | 330k | 130k | 490k | YES | NO |
| 21 | 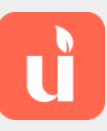 | iOS  & Android | 2017/03/01 | 2.0.1 | Information technology | NA | NA | NA | 12 | NA | YES | YES |
| 22 | 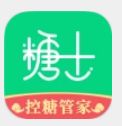 | iOS  & Android | 2018/05/01 | 2.5.1 | Information technology | 5 | 497 | 2,050k | 420k | 480k | YES | NO |
| 23 | 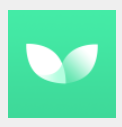 | iOS  & Android | 2018/02/21 | 3 | Health technology | 4.3 | 12 | NA | 3573 | 9 | NO | NO |
| 24 | 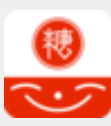 | Android | 2015/08/17 | 1.0 | Information technology | NA | NA | 1,805k | 227 | 303 | YES | YES |
| 25 | 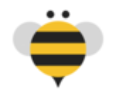 | Android | 2016/03/31 | 3.4.0 | Health technology | NA | NA | NA | 6661 | NA | NO | YES |
| 26 | 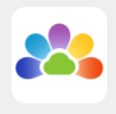 | iOS  & Android | 2018/06/28 | 4.4.4 | Information technology | 4.8 | 15 | 24k | 78k | 20k | YES | YES |
| 27 | 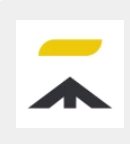 | iOS  & Android | 2018/08/15 | 6.7.1 | Information technology | 4 | 144 | NA | 480k | 1130k | YES | YES |
| 28 | 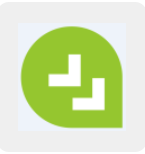 | iOS  & Android | 2018/08/06 | 3.3.2 | Science Institution | 4.7 | 12 | 150k | 12k | 180k | YES | YES |
| 29 | 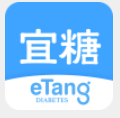 | iOS  & Android | 2018/06/01 | 3.1.6 | Health technology | 5 | 5 | 940 | 58k | 80k | NO | YES |
| 30 | 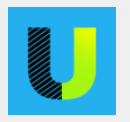 | Android | 2016/11/07 | 2.24.0 | Information technology | NA | NA | NA | 604 | NA | YES | NO |
| 31 | 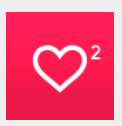 | iOS  & Android | 2018/02/28 | 2.2 | Health technology | 4 | 6 | 10k | 54k | 5,000 | YES | YES |
| 32 | 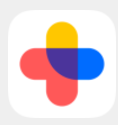 | iOS  & Android | 2017/03/01 | 3.0.8 | Health technology | 5 | 234 | 640k | 100k | NA | YES | YES |
| 33 | 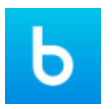 | Android | 2017/03/01 | 2.2.0 | Information technology | NA | NA | 10k | 3,461 | NA | NO | NO |
| 34 | 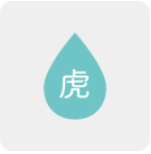 | iOS  & Android | 2017/11/01 | 1.1.1 | Information technology | 4.9 | 22 | NA | 38 | NA | YES | NO |
| 35 | 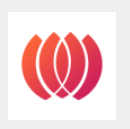 | Android | 2016/02/04 | 3.2 | Health technology | NA | NA | 4280 | 22k | 30k | YES | YES |
| 36 | 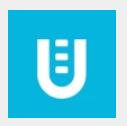 | iOS  & Android | 2018/02/28 | 3.3.1 | Health technology | 4.9 | 293 | 20k | 70k | 11k | YES | YES |
| 37 | 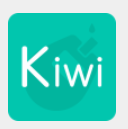 | Android | 2013/12/13 | 1.2.0 | Health technology | NA | NA | 2,562 | 10k | 10k | NO | NO |
| 38 | 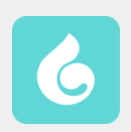 | iOS  & Android | 2018/02/01 | 2.0.3 | Health technology | 3.3 | 7 | NA | 1,765 | NA | YES | NO |
| 39 | 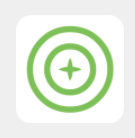 | Android | 2018/03/16 | 1.1.4 | Health technology | NA | NA | 136 | 139 | 46 | YES | YES |
| 40 | 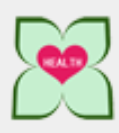 | Android | 2015/09/09 | 1.1.4 | Health technology | NA | NA | 1,907 | 874 | NA | NO | NO |
| 41 | 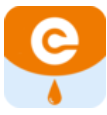 | Android | 2014/09/10 | 1.0.1.2 | Health technology | NA | NA | 374 | NA | NA | NO | YES |
| 42 | 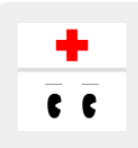 | iOS  & Android | 2017/08/09 | 0.9.1 | Health technology | NA | NA | 1,419 | 1,435 | NA | YES | YES |
| 43 | 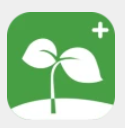 | iOS  & Android | 2016/12/13 | 3.5 | Information technology | NA | NA | 530 | 240 | NA | YES | YES |
| 44 | 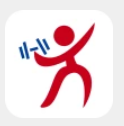 | iOS  & Android | 2018/02/13 | 7.6.0 | Health technology | 4.7 | 186 | 10k | 310k | 20k | YES | YES |
| 45 | 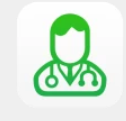 | Android | 2014/04/01 | 1.1.0 | Personal | NA | NA | 250 | NA | NA | NO | NO |
| 46 | 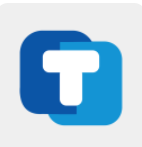 | iOS  & Android | 2018/09/01 | 4.3.0 | Health technology | 5 | 375 | 120k | 38k | 80k | YES | NO |
| 47 | 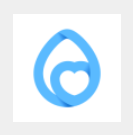 | iOS  & Android | 2018/03/01 | 1.1.2 | Health technology | NA | NA | NA | 214 | 97 | YES | YES |
| 48 | 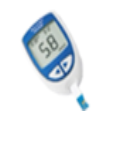 | Android | 2018/09/22 | 2.0 | Health technology | NA | NA | 8,408 | 137 | 1k | YES | YES |
| 49 | 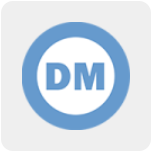 | Android | 2015/01/17 | 3.0.1 | Information technology | NA | NA | NA | NA | NA | NO | NO |
| 50 | 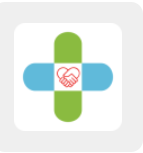 | Android | 2016/09/17 | 2.2.0 | Health technology | NA | NA | NA | 440 | NA | YES | NO |
| 51 | 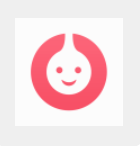 | iOS  & Android | 2018/08/01 | 5.3.5 | Health technology | NA | NA | 780k | 160k | 560k | YES | NO |
| 52 | 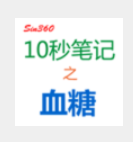 | Android | 2014/03/26 | 1.7 | Information technology | NA | NA | NA | 834 | NA | NO | NO |
| 53 | 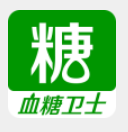 | Android | 2017/01/25 | 1.4.0 | Information technology | NA | NA | NA | 301 | NA | YES | YES |
| 54 | 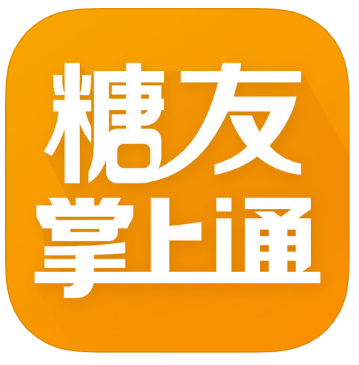 | iOS | 2018/01/01 | 1.0.1 | Health technology | NA | NA | NA | NA | NA | YES | NO |
| 55 | 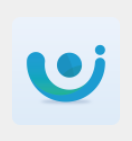 | iOS  & Android | 2018/03/02 | 2.2.8 | Health technology | 5 | 131 | NA | 62k | NA | YES | YES |
| 56 | 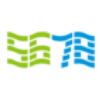 | Android | 2017/07/19 | 1.0.07 | Health technology | NA | NA | 106 | NA | NA | YES | YES |
| 57 | 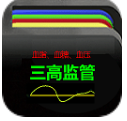 | Android | 2014/08/09 | 1.0.1 | Personal | NA | NA | NA | NA | 220 | NO | NO |
| 58 | 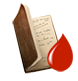 | Android | 2013/12/13 | 1.1 | Information technology | NA | NA | NA | 684 | 108 | NO | NO |
| 59 | 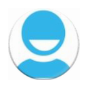 | Android | 2014/09/16 | 1.01 | Health technology | NA | NA | NA | NA | 270 | NO | NO |
| 60 | 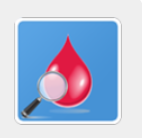 | Android | 2015/04/09 | 1.0 | Health technology | NA | NA | NA | 188 | NA | NO | NO |
| 61 | 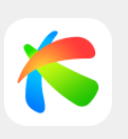 | iOS  & Android | 2017/08/01 | 4.6.1 | Health technology | NA | NA | NA | 160k | NA | YES | NO |
| 62 | 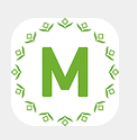 | iOS  & Android | 2018/02/01 | 1.0.3 | Health technology | 5 | 10 | NA | 36 | NA | YES | YES |
| 63 | 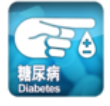 | Android | 2014/01/06 | 1.8.4 | Personal | NA | NA | 40k | 16k | 50k | NO | NO |
| 64 | 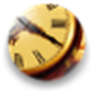 | Android | 2012/03/16 | 1.0 | Information technology | NA | NA | NA | NA | 1566 | NO | NO |
| 65 | 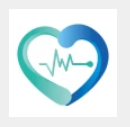 | iOS  & Android | 2015/04/22 | 1.0 | Health technology | NA | NA | 1,025 | 1,936 | 1,000 | YES | YES |
| 66 | 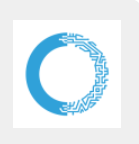 | iOS  & Android | 2017/09/01 | 2.0.1 | Information technology | NA | NA | 111 | 258 | NA | YES | YES |
| 67 | 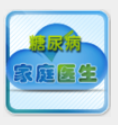 | Android | 2012/10/23 | 1.1 | Health technology | NA | NA | 5,222 | 913 | 5,000 | NO | NO |

Notes:

The information in this table was extracted by study raters during August-October, 2018 based on the information provided on the App markets or App descriptions.

¶Star rating and number of raters were extract from the iOS market if available. §Number of downloads were extracted from three Android markets if available.
