# Supplementary figures and images for: Quality, Functionality, and Features of Chinese Mobile Apps for Diabetes Self-Management: Systematic Search and Evaluation of Mobile Apps
Source: JMIR Mhealth Uhealth. 2020 Apr 7;8(4):e14836. doi: 10.2196/14836 (PMC7175187; doi:10.2196/14836)

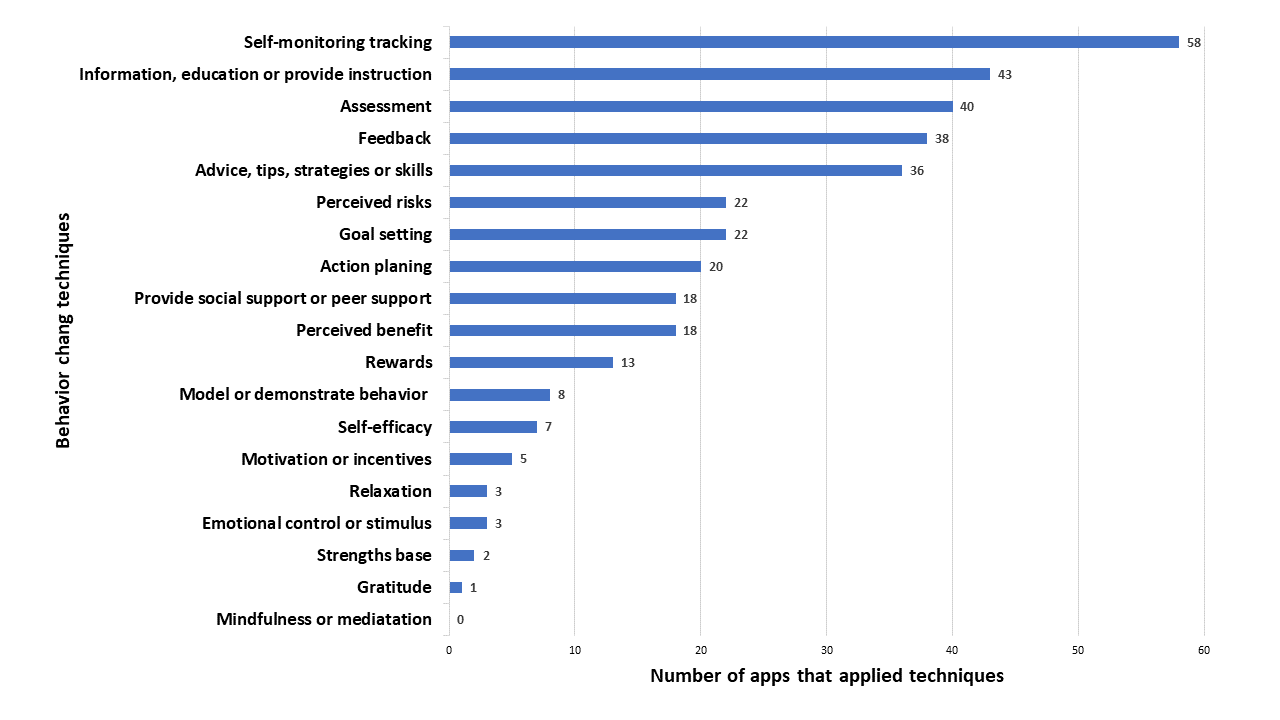

Supplement: Multimedia Appendix 2 [file mhealth_v8i4e14836_app2.png]
